# Bronchial Microbiota and the Stress Associated with Invasive Diagnostic Tests in Lung Cancer vs. Benign Pulmonary Diseases: A Cross-Sectional Study

**DOI:** 10.3390/diagnostics13142419

**Published:** 2023-07-20

**Authors:** Patricia Hogea, Emanuela Tudorache, Ovidiu Fira-Mladinescu, Camelia Pescaru, Diana Manolescu, Felix Bratosin, Ovidiu Rosca, Manaswini Kakarla, Florin George Horhat, Cristian Oancea

**Affiliations:** 1 Center for Research and Innovation in Precision Medicine of Respiratory Diseases, “Victor Babes” University of Medicine and Pharmacy, Eftimie Murgu Square 2, 300041 Timisoara, Romania; hogea.patricia@umft.ro (P.H.); tudorache_emanuela@yahoo.com (E.T.); pescaru.camelia@umft.ro (C.P.); dmanolescu@umft.ro (D.M.); oancea@umft.ro (C.O.); 2 Doctoral School, “Victor Babes” University of Medicine and Pharmacy, Eftimie Murgu Square 2, 300041 Timisoara, Romania; felix.bratosin@umft.ro; 3 Discipline of Radiology, Faculty of General Medicine, “Victor Babes” University of Medicine and Pharmacy Timisoara, Eftimie Murgu Square 2, 300041 Timisoara, Romania; 4 Discipline of Infectious Diseases, Faculty of General Medicine, “Victor Babes” University of Medicine and Pharmacy Timisoara, Eftimie Murgu Square 2, 300041 Timisoara, Romania; ovidiu.rosca@umft.ro; 5 Kamineni Institute of Medical Sciences, School of Medicine, Hyderabad 500001, India; manaswinikakarla@gmail.com; 6 Multidisciplinary Research Center on Antimicrobial Resistance (MULTI-REZ), Microbiology Department, “Victor Babes” University of Medicine and Pharmacy, 300041 Timisoara, Romania

**Keywords:** lung cancer, bronchial lavage, microbial analysis, hospital anxiety, depression

## Abstract

Lung cancer is the leading cause of cancer-related deaths worldwide. This study aimed to compare the bronchial microbiota of patients with lung cancer and patients with benign pulmonary diseases undergoing bronchoscopy, and to assess the stress levels associated with invasive diagnostic lung tests. A cross-sectional study was conducted at the “Victor Babes” Hospital for Infectious Diseases and Pulmonology in Timisoara, Romania. A total of 33 patients with histologically diagnosed bronchopulmonary cancer and 33 control patients with benign lung pathologies underwent bronchoscopy. Bronchial microbiota was analyzed by multiplex PCR, culture media, and cytology. Anxiety and depression levels were assessed using the ECOG performance status scale, Karnofsky scale, GAD-7, PHQ-9, and HADS questionnaires. There were no significant differences in the presence of common microbial species between the two groups, except for *Acinetobacter* spp. Which was identified in 15.2% of patients with lung cancer and 0.0% in the control group, *Candida* spp. Was more prevalent in the benign group (24.2% vs. 6.1%), and the Parainfluenza virus was detected only in the malignant group (21.1% vs. 0.0%). Cytology results showed a higher prevalence of atypical and tumoral cells in the malignant group (39.4% and 30.0%, respectively), as well as higher lymphocyte levels in the benign group (69.7% vs. 24.2%). Patients with lung cancer had significantly lower performance status on the ECOG scale (2.34 vs. 1.92), lower Karnofsky scores (71.36 vs. 79.43), and higher GAD-7 and PHQ-9 scores at the initial evaluation compared to the benign group. At the 90-day follow-up, ECOG and Karnofsky scores remained significantly different from the initial evaluation, but only GAD-7 scores showed a significant difference between the two groups. There were differences in the bronchial microbiota between patients with lung cancer and benign pulmonary diseases, with a higher prevalence of *Candida* spp. in the benign group and exclusive detection of *Acinetobacter* spp. and Parainfluenza virus in the malignant group. Patients with lung cancer exhibited higher stress levels, more severe anxiety, and depression symptoms, which persisted during follow-up. Further research is needed to understand the role of bronchial microbiota in lung cancer and the impact of stress on patient outcomes.

## 1. Introduction

Lung cancer is a leading cause of cancer-related mortality worldwide, accounting for approximately 1.8 million deaths annually, as reported by Sung et al. (2021) [1]. Despite advances in diagnostics and therapeutics, the overall survival rate for lung cancer patients remains poor, primarily due to late-stage diagnosis and limited treatment options [2,3]. Chronic lung diseases, such as chronic obstructive pulmonary disease (COPD) and interstitial lung disease (ILD), are also significant contributors to morbidity and mortality, posing a considerable burden on healthcare systems and society [4,5,6].

Invasive diagnostic procedures, including bronchoscopy, are frequently employed to diagnose and manage both malignant and benign pulmonary diseases. Bronchoscopy is a well-established technique that allows for direct visualization of the bronchial tree, biopsy of lung tissue, and sampling of bronchial secretions [7,8]. However, undergoing an invasive procedure can be a source of considerable stress and anxiety for patients, leading to potential psychological repercussions [9,10]. As such, there is a need to better understand the association between bronchoscopy and patients’ stress levels.

Recent advances in microbiome research have led to a growing appreciation of the role that the lung microbiota may play in respiratory health and disease [11,12]. The lung microbiota is a complex ecosystem of microorganisms, including bacteria, fungi, and viruses, which are thought to contribute to pulmonary homeostasis and pathogenesis [13]. Dysbiosis, or an imbalance in the composition of the lung microbiota, has been implicated in the development and progression of various pulmonary diseases, including lung cancer and COPD [14,15]. Analyzing the bronchial microbiota in patients with lung cancer and benign pulmonary diseases may help elucidate potential biomarkers for early diagnosis and therapeutic targets for personalized interventions [16].

Moreover, there is growing evidence to suggest that the gut-lung axis, a bidirectional communication network linking the gut and lung microbiomes, may influence stress and anxiety levels in patients [17]. For instance, stress-induced alterations in the gut microbiota have been shown to affect the lung microbiota and vice versa, with potential implications for pulmonary disease pathogenesis and the patient’s psychological well-being [18]. As such, understanding the interaction between the bronchial microbiota and patients’ stress levels could provide valuable insights into the development of strategies to mitigate anxiety and improve the overall quality of life for those undergoing invasive lung tests.

Hospitalization and invasive diagnostic procedures can be a source of considerable distress for patients, leading to the development of anxiety and depression [19]. The hospital environment, characterized by unfamiliar surroundings, loss of personal autonomy, and fear of the unknown, can trigger psychological symptoms and exacerbate pre-existing mental health conditions [20]. Additionally, patients with lung cancer and chronic lung diseases may experience increased levels of anxiety and depression due to the uncertainty of their prognosis and the potential impact of their illness on their daily lives [21].

The primary aim of this study is to conduct a comparative analysis of the bronchial microbiota in patients with lung cancer and benign pulmonary diseases undergoing bronchoscopy to help illuminate the potential role of the bronchial microbiota in disease pathogenesis and the possible interplay between microbiota and patients’ psychological well-being. Furthermore, this study seeks to assess the stress levels associated with invasive lung tests in patients with lung cancer and benign pulmonary diseases. By evaluating the prevalence of anxiety and depression in these patients, we aim to better understand the psychological impact of invasive diagnostic procedures and the hospital environment on their mental health. This understanding will be instrumental in developing targeted interventions to mitigate anxiety and improve patients’ overall well-being during their hospital stay and beyond.

## 2. Materials and Methods

### 2.1. Study Design

A cross-sectional study was designed at the “Victor Babes” Hospital for Infectious Diseases and Pulmonology in Timisoara, Romania. The study was conducted according to the guidelines of the Declaration of Helsinki. The researchers involved in the current study gathered background and medical data from the hospital database and the associated patients’ paper records, where all treatments, procedures, and demographics were registered. The study protocol was approved on 23 September 2022, with the number 10218.

Patients admitted to the Pneumology Clinic of the “Victor Babes” Clinical Hospital from Timisoara between September 2022 and February 2023 were included in this study. The inclusion criteria comprised: (1) patients with a major suspicion of bronchopulmonary cancer on the chest computed tomography (CT), (2) the need to perform bronchoscopy for diagnostic purposes, (3) patients’ agreement for exploratory lung interventions and participation in the current study, and (4) participation at the 90-day follow-up. Exclusion criteria comprised a lack of consent and incomplete survey results, as well as not participating in the follow-up study.

Case-matching was performed with a 1:1 ratio between patients with lung cancer and those with benign lung disease based on age, gender, and history of exposure to respiratory hazards. At the end of the study period, 33 patients with histologically diagnosed bronchopulmonary cancer were included in the study, and 33 patients were included in the control group with the following pathologies: asthma, chronic bronchitis, emphysema, hypersensitivity pneumonitis, and interstitial lung disease. All patients and controls underwent bronchoscopy for diagnostic purposes before initiation of treatment, while microbial identification studies by multiplex PCR and conventional cultures were performed in all cases involved. All bronchoscopies were performed by one researcher, based on the hospital guidelines, in the same hospital unit.

Bronchoalveolar lavage (BAL) fluid obtained during bronchoscopy was used for microbial culture. Immediately after collection, BAL samples were transported to the microbiology laboratory. A portion of each sample was used for direct microscopic examination with Gram staining, and another portion was inoculated onto appropriate culture media to isolate aerobic and anaerobic bacteria. In addition to culture methods, multiplex PCR was employed for the detection and identification of microbial species in the BAL fluid. The process began with the extraction of total genomic DNA from the BAL samples. Commercially available kits were used for DNA extraction, following the manufacturer’s instructions. The extracted DNA was used as a template for the multiplex PCR. For our study, the choice of primers was based on the sequences of the 16S rRNA gene, which is highly conserved among bacteria and thus frequently used for their identification.

The variables considered for inclusion in the current study comprised (1) background data: age, body mass index, gender, smoking status, pack-year smoking, and exposure to respiratory hazards; (2) clinical data and investigations: signs and symptoms upon initial examination, duration since symptom onset, spirometry findings, degree of respiratory dysfunction; (3) bronchoalveolar lavage (BAL) fluid analysis: multiplex PCR assay, culture media, and cytology; (4) scales and survey results: ECOG, Karnofsky, GAD-7, PHQ-9, and HADS.

### 2.2. Interventions and Definitions

Patients included in the study were evaluated by two of the participating researchers in a blinded approach, or self-evaluated based on the questionnaire used. Functional status was examined using the ECOG performance status scale and the Karnofsky scale, while the levels of anxiety and depression were assessed by the GAD-7 and PHQ9 screening tools and the HADS scale.

The ECOG (Eastern Cooperative Oncology Group) performance status scale is a standardized tool used to assess the functional status of cancer patients. It is commonly used in clinical trials and in the practice of oncology to help determine appropriate treatment options [22]. The ECOG scale ranges from 0 to 5, with 0 representing normal activity and no symptoms and 5 representing death. The scale is divided into five categories: ECOG 0: Fully active, able to carry out all pre-disease performance without restriction. ECOG 1: Restricted in physically strenuous activity but ambulatory and able to carry out work of a light or sedentary nature, such as light housework or office work. ECOG 2: Ambulatory and capable of all self-care but unable to carry out any work activities. Up and about more than 50% of waking hours. ECOG 3: Capable of only limited self-care, confined to bed or chair more than 50% of waking hours; ECOG 4: Completely disabled and unable to carry on any self-care, or totally confined to bed or chair. The ECOG performance status scale is often used by healthcare providers to determine the appropriate treatment plan for cancer patients, as patients with lower ECOG scores generally have a better prognosis and may be able to tolerate more aggressive treatments.

GAD-7 (Generalized Anxiety Disorder-7) is a self-reported screening tool used to assess the severity of anxiety symptoms in adults [23]. The GAD-7 tool consists of seven questions that ask about common anxiety symptoms experienced over the past two weeks. Each question is rated on a scale of 0 to 3, with 0 being “not at all” and 3 being “nearly every day.” The total score on the GAD-7 screening tool can range from 0 to 21, with higher scores indicating more severe anxiety symptoms. A score of 5 or higher is typically considered a positive screen for generalized anxiety disorder (GAD), but the cutoff may vary depending on the context in which the scale is used. GAD-7 is a useful tool for identifying individuals who may benefit from further evaluation and treatment for anxiety disorders. It is also commonly used in research studies to measure the severity of anxiety symptoms and to track changes in symptoms over time.

The PHQ-9 (Patient Health Questionnaire-9) is a self-reported screening tool used to assess the severity of depression symptoms in adults, built in a similar manner to the GAD-7 scale [24]. The PHQ-9 consists of nine questions that ask about common depression symptoms experienced over the past two weeks. Each question is rated on a scale of 0 to 3, with 0 being “not at all” and 3 being “nearly every day.” The total score on the PHQ-9 can range from 0 to 27, with higher scores indicating more severe depression symptoms. A score of 10 or higher is typically considered a positive screen for depression, but the cutoff may vary depending on the context in which the scale is used.

The Karnofsky Performance Status Scale is a widely used tool for measuring the functional status and ability of individuals with cancer, chronic illness, or undergoing treatment [25]. It was developed by Dr. David A. Karnofsky in the 1940s and has since been adopted by healthcare providers across multiple fields. The Karnofsky scale measures functional status on a scale of 0–100% with increments of 10%. The higher the score, the better the individual’s ability to carry out activities of daily living. The scores are assigned based on an individual’s ability to perform the following tasks:

*100%: Normal, no complaints, no evidence of disease*
*90%: Able to carry on normal activities; minor signs or symptoms of disease.*
*80%: Able to carry on normal activities with some difficulty; signs or symptoms of disease.*
*70%: Cares for self; unable to carry on normal activities or to do active work.*
*60%: Requires occasional assistance but is able to care for most of their personal needs.*
*50%: Requires considerable assistance and frequent medical care.*
*40%: Disabled; requires special care and assistance.*
*30%: Severely disabled; hospitalization is indicated, but death is not imminent.*
*20%: Very sick; hospitalization necessary; active supportive treatment necessary.*
*10%: Moribund; fatal processes progressing rapidly.*

The Hospital Anxiety and Depression Scale (HADS) is a self-report scale designed to measure anxiety and depression in individuals who are being treated in a hospital or outpatient setting. It is a widely used screening tool for identifying individuals with anxiety and depression symptoms. The HADS consists of 14 items, seven of which assess anxiety symptoms (HADS-A) and seven of which assess depression symptoms (HADS-D) [26]. Each item is scored on a 4-point scale, with higher scores indicating greater levels of anxiety or depression. The HADS does not include questions about physical symptoms, which helps to distinguish anxiety and depression from physical illness.

### 2.3. Statistical Analysis

The IBM SPSS v.27 statistical software and Microsoft Excel were used for statistical analysis and data representation. The Kolmogorov–Smirnov test was used to assess the normality of the data. The mean value, which represents central tendency, and the standard deviation, which measures dispersion, were used to represent normally distributed data. A Student’s *t*-test was used to examine the difference in means between the two comparison groups. The median and interquartile range (IQR) were used to characterize non-normally distributed data, presented in box plots, while the Mann–Whitney U test was used to compare these variables. Considering the frequency assumption for the Chi-square test was not fulfilled, proportions were compared using Fisher’s exact test. To address the possible interplay between microbiota and patients’ psychological well-being, we ran a regression analysis for each microbe and the PHQ results. A *p*-value below 0.05 was regarded as statistically significant.

## 3. Results

### Patients’ Demographics

Table 1 presents the background data of study participants, including demographics, BMI, and smoking history, for two groups: malignant (*n* = 33) and benign (*n* = 33). The malignant group consists of patients with lung cancer, while the benign group is composed of patients with chronic lung disease. The mean age of the malignant group is 62.7 ± 8.7 years, with an age range of 48–75 years, while the mean age of the benign group is 58.2 ± 13.6 years, with an age range of 37–73 years. The difference in mean age between the two groups is not statistically significant (*p* = 0.114), suggesting that age is not a distinguishing factor between the two groups. The mean BMI in the malignant group is 23.5 ± 4.1 kg/m^2^, which is significantly lower than that of the benign group (27.3 ± 5.8 kg/m^2^), with a *p*-value of 0.003. When evaluating BMI categories, there is a statistically significant difference between the groups (*p* = 0.030). In the malignant group, 54.5% have a BMI of 25–29.9 kg/m^2^, and 39.4% have a BMI greater than 30 kg/m^2^, while in the benign group, these percentages are 24.2% and 57.6%, respectively.

Gender distribution differs between the two groups, with a higher percentage of males in the malignant group (63.6%) than in the benign group (42.4%), but the difference is not statistically significant (*p* = 0.084). The percentage of smokers or ex-smokers is higher in the malignant group (60.6%) compared to the benign group (48.5%), but this difference is also not statistically significant (*p* = 0.322). However, there is a significant difference in pack-year smoking history between the two groups (*p* < 0.001), with a median of 34.0 pack-years (interquartile range 22.5–41.0) in the malignant group, compared to a median of 18.5 pack-years (interquartile range 12.0–29.5) in the benign group. This indicates that the malignant group has more exposure to smoking than the benign group. Finally, exposure to respiratory hazards is comparable between the two groups, with 42.4% of the malignant group and 51.5% of the benign group reporting exposure, and no statistically significant difference was observed (*p* = 0.459).

Table 2 presents the clinical data and investigation results of study participants, comparing patients with lung cancer and those with chronic lung disease. In terms of clinical data, the prevalence of coughing is similar between the two groups (87.9% in the malignant group and 78.8% in the benign group), with no statistically significant difference (*p* = 0.321). The percentage of dry cough also shows no significant difference between the groups (69.0% in the malignant group and 65.4% in the benign group, *p* = 0.777). Thoracic pain is more common in the malignant group (30.3%) than the benign group (6.1%), with a statistically significant difference (*p* = 0.010). Hemoptysis, fever, and dyspnea show no significant differences between the groups (*p* = 0.281, *p* = 0.302, and *p* = 0.353, respectively).

Weight loss was significantly more prevalent in the malignant group (69.7%) compared to the benign group (6.1%, *p* < 0.001). In contrast, anorexia was exclusively observed in the benign group (54.5%) and absent in the malignant group, with a highly significant difference (*p* < 0.001). Fatigue was reported by 90.9% of the malignant group and 78.8% of the benign group, with no significant difference (*p* = 0.969). Wheezing and stridor were more prevalent in the benign group (51.5%) compared to the malignant group (15.2%), with a significant difference (*p* = 0.002). Pulmonary auscultation results showed no significant difference between the groups (*p* = 0.083). The mean duration of symptom onset is significantly shorter in the malignant group (5.6 ± 3.7 months) compared to the benign group (15.2 ± 10.4 months, *p* < 0.001). For spirometry investigation results, there was a significant difference in the distribution of patterns (*p* < 0.001). The malignant group showed a higher prevalence of obstructive (33.3%) and mixed patterns (42.4%), while the benign group had a higher prevalence of restrictive patterns (45.5%). Regarding the degree of respiratory dysfunction (FEV1), there was no significant difference between the groups (*p* = 0.173).

The bronchoalveolar lavage fluid analysis presented in Table 3 includes findings from a multiplex PCR assay, culture media, and cytology. In the multiplex PCR assay, there was no significant difference in the presence of commensal flora, *Pseudomonas aeruginosa*, *Streptococcus* spp., *Klebsiella* spp., *Staphylococcus aureus*, or *Escherichia coli* between the malignant and benign groups (*p*-values ranging from 0.076 to 0.741). On the multiplex PCR assay, *Acinetobacter* spp. was identified in a significantly higher proportion of patients with lung cancer (15.2% vs. 0.0%). In addition, there was a significant difference in the presence of *Candida* spp. and Parainfluenza virus between the two groups. *Candida* spp. was more prevalent in the benign group (24.2%) compared to the malignant group (6.1%, *p* = 0.039). Parainfluenza virus was only detected in the malignant group (12.1%) and not found in the benign group (*p* = 0.039). In the culture media analysis, there was no significant difference in the presence of commensal flora, *Pseudomonas aeruginosa*, *Streptococcus* spp., *Acinetobacter* spp., *Klebsiella* pneumoniae, *Staphylococcus aureus*, or *Escherichia coli* between the malignant and benign groups (*p*-values ranging from 0.150 to 1). The presence of *Candida* spp. was not significantly different between the malignant group (9.1%) and the benign group (21.2%, *p* = 0.169).

Cytology results showed significant differences in the presence of atypical cells, tumoral cells, and lymphocytes between the two groups. Atypical cells were more prevalent in the malignant group (39.4%) compared to the benign group (12.1%, *p* = 0.011). Tumoral cells were found exclusively in the malignant group (30.3%) and were not detected in the benign group, with a highly significant difference (*p* < 0.001). Lymphocytes are more prevalent in the benign group (69.7%) compared to the malignant group (24.2%, *p* < 0.001). There was no significant difference in the presence of neutrophils between the groups (*p* = 0.121), while eosinophils showed a significant difference, being more prevalent in the benign group (51.7%) compared to the malignant group (36.4%, *p* = 0.010).

Table 4 presents the scale and survey results comparing patients with lung cancer and those with chronic lung disease at both the initial and follow-up time points. The scales and surveys include the Eastern Cooperative Oncology Group (ECOG) performance status, Karnofsky scale, General Anxiety Disorder-7 (GAD-7), and Patient Health Questionnaire-9 (PHQ-9). At the initial time point, the malignant group had a significantly higher mean ECOG score (2.34 ± 0.66) compared to the benign group (1.92 ± 0.24, *p* = 0.001), indicating a lower performance status. The mean Karnofsky score was significantly lower in the malignant group (71.36 ± 8.68) compared to the benign group (79.34 ± 6.18, *p* < 0.001), suggesting a poorer functional status. The mean GAD-7 score was significantly higher in the malignant group (7.18 ± 2.41) compared to the benign group (5.45 ± 3.52, *p* = 0.023), indicating more severe anxiety symptoms. The mean PHQ-9 score was also significantly higher in the malignant group (4.90 ± 2.29) than in the benign group (3.45 ± 2.84, *p* = 0.026), implying more severe depression symptoms.

**Figure 1:**
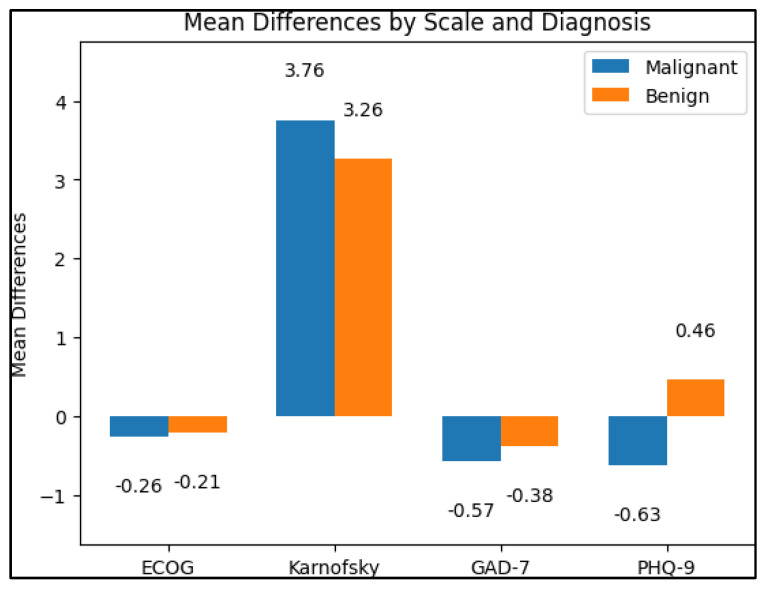
Scale and survey results: mean difference between the 90-day follow-up and the initial values.

At the follow-up time point, the malignant group continued to have a significantly higher mean ECOG score (2.08 ± 0.52) than the benign group (1.71 ± 0.39, *p* = 0.002). The mean Karnofsky score remained significantly lower in the malignant group (75.12 ± 8.07) compared to the benign group (82.60 ± 6.49, *p* < 0.001). The mean GAD-7 score remained significantly higher in the malignant group (6.61 ± 2.25) compared to the benign group (5.07 ± 3.14, *p* = 0.035). However, there was no significant difference in the mean PHQ-9 score between the malignant group (4.27 ± 2.06) and the benign group (3.91 ± 1.95, *p* = 0.468) at the follow-up time point, as presented in Figure 1.

**Figure 2:**
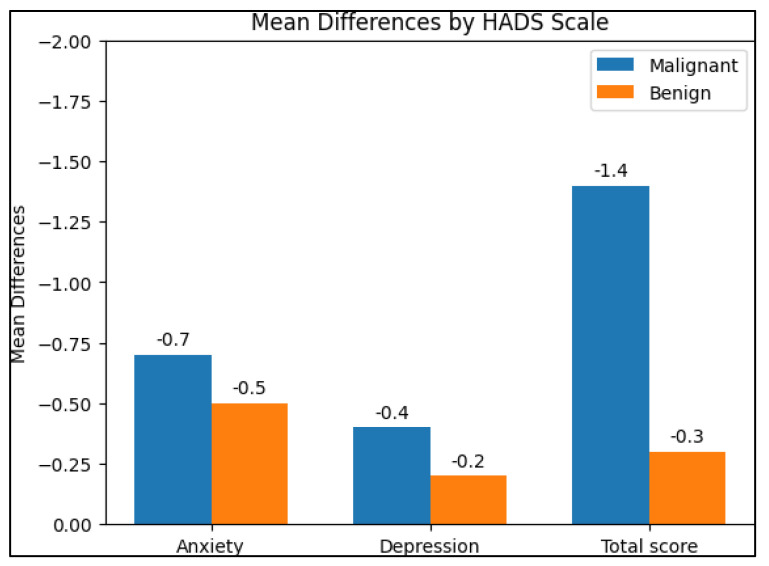
Analysis of HADS questionnaire results (higher scores indicate more severe anxiety or depressive symptoms); mean difference between the 90-day follow-up and the initial values.

Table 5 presents the results of the Hospital Anxiety and Depression Scale (HADS) questionnaire for patients with lung cancer and those with chronic lung disease at the time of diagnosis and at follow-up. The HADS questionnaire measures levels of anxiety and depression, with higher scores indicating greater levels of anxiety or depression. At the time of diagnosis, the mean anxiety score in the malignant group was 7.6 ± 4.1, while the benign group had a mean score of 6.7 ± 3.5. The difference between the two groups was not statistically significant (*p* = 0.341). In contrast, the mean depression score was 7.2 ± 3.6 in the malignant group and 5.3 ± 2.3 in the benign group, with a statistically significant difference between the two groups (*p* = 0.013). The total HADS score was significantly different between the malignant group (12.8 ± 6.3) and the benign group (10.1 ± 4.2, *p* = 0.044), as presented in Figure 2.

At follow-up, the mean anxiety score for the malignant group was 6.9 ± 4.7, and the benign group had a mean score of 6.2 ± 4.5. No statistically significant difference was found between the groups (*p* = 0.538). The mean depression score was 6.8 ± 3.1 in the malignant group and 5.1 ± 4.8 in the benign group, with no significant difference between the groups (*p* = 0.092). The total HADS score was not significantly different between the malignant group (11.4 ± 5.5) and the benign group (9.8 ± 6.1, *p* = 0.267). Lastly, there was no significant association found between bronchial microbiota and depression, as measured on the PH-9 survey (Table 6).

## 4. Discussion

### 4.1. Important Findings

In this cross-sectional study, we aimed to compare the bronchial microbiota between patients with lung cancer and those with benign pulmonary diseases undergoing bronchoscopy, as well as to evaluate the stress levels associated with invasive lung tests. The findings revealed significant differences between the malignant and benign groups found in BMI, BMI categories, and pack-year smoking history, suggesting that BMI and smoking exposure may be relevant factors in differentiating between patients with lung cancer and those with chronic lung disease. In addition, significant differences between the malignant and benign groups were observed in thoracic pain, weight loss, anorexia, wheezing and stridor, symptom onset duration, and spirometry patterns. These findings may aid in differentiating between patients with lung cancer and those with chronic lung disease based on their clinical data and investigation results.

Another important finding was that significant differences in bronchoalveolar lavage fluid analysis between the malignant and benign groups were found regarding the presence of *Candida* spp., Parainfluenza virus, atypical cells, tumoral cells, lymphocytes, and eosinophils. Regarding the surveys and questionnaires, at both the initial and follow-up time points, patients with lung cancer exhibited significantly lower performance and functional status, as well as more severe anxiety symptoms compared to those with chronic lung disease. At the initial measurement, the malignant group had more severe depression symptoms, but this difference was not significant at the follow-up time point. Lastly, the HADS questionnaire results revealed that at the time of diagnosis, patients with lung cancer had significantly higher depression and total scores compared to those with chronic lung disease. However, at follow-up, there were no statistically significant differences in anxiety, depression, or total scores between the two groups.

One of the key findings of this study was the distinct differences in bronchial microbiota between lung cancer patients and those with benign pulmonary diseases. Although there were no significant differences in the presence of common microbial species between the two groups, some noteworthy observations were made. *Acinetobacter* spp. was identified in 15.2% of lung cancer patients and was absent in the control group. *Acinetobacter* spp. is a Gram-negative bacterium that has been previously associated with nosocomial infections, and some studies have suggested a potential link between pathogenic bacteria such as *Acinetobacter* spp. and lung cancer development or progression [27,28]. Further studies are needed to explore the potential role of *Acinetobacter* spp. in lung cancer pathogenesis. *Candida* spp. was found to be more prevalent in the benign group (24.2% vs. 6.1%).

Although *Candida* spp. is primarily recognized as a commensal microorganism in the human body, some studies have suggested that it may be correlated with certain respiratory diseases, such as asthma and chronic obstructive pulmonary disease, or associated with the treatment used for these chronic lung diseases [29,30]. The higher prevalence of *Candida* spp. in the benign group might be associated with the underlying lung pathologies in this group, warranting further investigation. Additionally, the Parainfluenza virus was exclusively detected in the malignant group (21.1% vs. 0.0%). Parainfluenza virus is a known respiratory pathogen that has been associated with various respiratory diseases, including pneumonia and bronchiolitis [31]. However, although certain viral infections are linked with carcinogenesis, the parainfluenza virus was not described as such [31,32].

The role of pathogenic bacteria in lung cancer has been an emerging area of research in recent years. It is now widely recognized that the lung microbiota, which is composed of diverse bacterial species, plays a crucial role in maintaining lung health [33]. Disturbances in the composition of the lung microbiota, known as dysbiosis, have been linked to the development and progression of various lung diseases, including lung cancer [34]. For example, some studies have reported an increased prevalence of potentially pathogenic bacteria, such as *Streptococcus* spp., *Staphylococcus* spp., and *Pseudomonas aeruginosa*, in the lungs of lung cancer patients compared to healthy controls [35,36].

There are several potential mechanisms through which pathogenic bacteria may contribute to lung cancer development and progression, such as the production of carcinogenic substances that include reactive oxygen species, reactive nitrogen species, and genotoxins, which can cause DNA damage and genomic instability, eventually leading to the initiation of lung cancer [37,38]. Chronic inflammation is a well-established risk factor for lung cancer, while pathogenic bacteria can induce a chronic inflammatory response in the lung by activating immune cells and releasing pro-inflammatory cytokines, chemokines, and other mediators that may promote tumor growth, angiogenesis, and metastasis [39]. Pathogenic bacteria can also modulate the host immune response by altering the balance between pro- and anti-inflammatory cytokines, impairing the function of immune cells, or promoting the expansion of immunosuppressive cell populations [40].

Cytological analysis of bronchoalveolar lavage (BAL) fluid revealed a higher prevalence of atypical and tumoral cells in the malignant group (39.4% and 30.0%, respectively), which is consistent with the histological diagnosis of bronchopulmonary cancer in these patients. The higher lymphocyte levels observed in the benign group (69.7% vs. 24.2%) may be indicative of the underlying inflammatory processes associated with benign lung pathologies. Lymphocytic infiltration has been reported in various benign pulmonary diseases, such as asthma, COPD, and interstitial lung diseases [41]. The differences in cytological findings and lymphocyte levels between the two groups highlight the potential diagnostic and prognostic value of BAL cytology in distinguishing lung cancer from benign pulmonary diseases.

Lung cancer patients in this study exhibited significantly higher stress levels, more severe anxiety, and more depression symptoms than the benign group. The significantly lower ECOG performance status scale scores (2.34 vs. 1.92) and Karnofsky scores (71.36 vs. 79.43) in the lung cancer group suggest poorer functional status in these patients. Higher GAD-7 and PHQ-9 scores at the initial evaluation further highlight the increased levels of anxiety and depression in lung cancer patients. During follow-up, ECOG and Karnofsky scores remained significantly different from the initial evaluation, but only GAD-7 scores showed a significant difference between the two groups. This persistence of higher stress levels and anxiety in lung cancer patients might be attributed to various factors, including the burden of the cancer diagnosis, fear of treatment outcomes, and concerns about the prognosis. The psychological impact of lung cancer has been well documented in the literature, with several studies reporting a high prevalence of anxiety and depression among lung cancer patients [42,43]. The association between stress levels and patient outcomes is a topic of growing interest in the field of oncology. It has been suggested that stress may contribute to the progression of cancer through various mechanisms, such as immune suppression, increased inflammation, and alterations in cellular processes [44]. However, the exact impact of stress on lung cancer outcomes remains unclear and warrants further investigation.

Even though the observed differences from the surveys did not affect the process of diagnosing and treating patients, our study has several implications for clinical practice and future research. First, the observed differences in bronchial microbiota between lung cancer patients and those with benign pulmonary diseases may provide valuable insights into the potential role of microbiota in lung cancer development and progression. Further studies are needed to explore the mechanistic links between specific microbial species and lung cancer pathogenesis, which may eventually lead to the identification of novel therapeutic targets or diagnostic biomarkers. Second, the higher stress levels, anxiety, and depression symptoms in lung cancer patients underscore the importance of addressing psychological well-being in the management of these patients. Adequate screening for psychological distress and timely provision of supportive care services, such as counseling or psychotherapy, may help improve the quality of life and potentially even influence the clinical outcomes of lung cancer patients.

### 4.2. Study Limitations

Although this study provides valuable insights into the bronchial microbiota of patients with lung cancer and benign pulmonary diseases, there are some limitations to consider. First, the sample size of 33 patients in each group may be too small to detect subtle differences in microbial composition and stress levels between the groups. Second, the study is cross-sectional in nature, which limits the ability to establish causality or observe changes over time. Third, the study was conducted at a single hospital, which may limit the generalizability of the findings to other settings or populations. Fourth, bronchoscopies were performed by only one researcher, which may introduce potential bias or variability in the procedure. Moreover, the pack-year median was significantly higher in the malignant group, which might be a confounding factor, as smoking can influence airway microbiota [45]. Lastly, self-reported measures such as the GAD-7, PHQ-9, and HADS scales are subject to response bias, and patients may underreport or overreport their symptoms. Despite these limitations, the study offers important information that can inform future research and contribute to the understanding of bronchial microbiota and stress levels in patients undergoing invasive lung tests. Moreover, future studies could include a control group of individuals with no lung pathology to determine the true difference between a healthy person and those with different lung pathologies.

## 5. Conclusions

In conclusion, this study revealed differences in the bronchial microbiota between patients with lung cancer and those with benign pulmonary diseases undergoing bronchoscopy. A higher prevalence of *Candida* spp. was observed in the benign group, while *Acinetobacter* spp. and Parainfluenza virus were exclusively detected in the malignant group. Furthermore, patients with lung cancer exhibited higher stress levels, more severe anxiety, and more depression symptoms compared to the benign group. These differences persisted during follow-up evaluations. Notably, the ECOG and Karnofsky scores for the malignant group were significantly lower, indicating poorer performance status. The findings of this study emphasize the importance of further research to better understand the role of bronchial microbiota in lung cancer development and progression, as well as the impact of stress, anxiety, and depression on patient outcomes in the context of invasive lung tests.

## Figures and Tables

**Table 1:** Background data of study participants.

Variables	Malignant (*n* = 33)	Benign (*n* = 33)	*p*-Value
Age (mean ± SD)	62.7 ± 8.7	58.2 ± 13.6	0.114
Age range	48–75	37–73	–
BMI (mean ± SD)	23.5 ± 4.1	27.3 ± 5.8	0.003
**BMI categories**			0.030
18.5–24.9 (kg/m^2^)	2 (6.1%)	6 (18.2%)	
25–29.9 (kg/m^2^)	18 (54.5%)	8 (24.2%)	
>30 (kg/m^2^)	13 (39.4%)	19 (57.6%)	
Gender (male, %)	21 (63.6%)	14 (42.4%)	0.084
Smoker/Ex-smoker (yes, %)	20 (60.6%)	16 (48.5%)	0.322
Pack-year smoking (median, IQR)	34.0 (22.5–41.0)	18.5 (12.0–29.5)	<0.001
Exposure to respiratory hazards (yes, %)	14 (42.4%)	17 (51.5%)	0.459
Benign lung disease			
Chronic bronchitis	-	4 (12.1%)	-
Emphysema	-	9 (27.3%)	-
ILD	-	4 (12.1%)	-
HP	-	8 (24.2%)	-
Others	-	6 (18.2%)	-
Cancer types		2 (6.1%)	
SCLC	14 (42.4%)	-	-
NSCLC	19 (57.6%)	-	-

BMI—Body Mass Index; SD—Standard Deviation; IQR—Interquartile Range; ILD—Interstitial Lung Disease; HP—Hypersensitivity Pneumonitis; SCLC—Small Cell Lung Cancer; NSCLC—Non-Small Cell Lung Cancer.

**Table 2:** Clinical data and investigations.

Variables	Malignant (*n* = 33)	Benign (*n* = 33)	*p*-Value
**Clinical data**			
Cough (*n*, %)	29 (87.9%)	26 (78.8%)	0.321
Type of cough (dry, %)	20 (69.0%)	17 (65.4%)	0.777
Thoracic pain (*n*, %)	10 (30.3%)	2 (6.1%)	0.010
Hemoptysis (*n*, %)	6 (18.2%)	3 (9.1%)	0.281
Fever (*n*, %)	1 (3.0%)	3 (9.1%)	0.302
Weight loss (*n*, %)	23 (69.7%)	2 (6.1%)	<0.001
Dyspnea (*n*, %)	28 (84.8%)	25 (75.8%)	0.353
Anorexia (*n*, %)	0 (0.0%)	18 (54.5%)	<0.001
Fatigue (*n*, %)	30 (90.9%)	26 (78.8%)	0.969
Wheezing and stridor (*n*, %)	5 (15.2%)	17 (51.5%)	0.002
Pulmonary auscultation (normal, %)	18 (54.5%)	11 (33.3%)	0.083
Symptom onset, months (mean ± SD)	5.6 ± 3.7	15.2 ± 10.4	<0.001
**Investigations**			
**Spirometry**			<0.001
Normal	5 (15.2%)	9 (27.3%)	
Obstructive pattern	11 (33.3%)	6 (18.2%)	
Restrictive pattern	3 (9.1%)	15 (45.5%)	
Mixt pattern	14 (42.4%)	3 (9.1%)	
**Degree of respiratory dysfunction (FEV1)**			0.173
Mild (≥80)	13 (39.4%)	18 (54.5%)	
Moderate (50–79)	15 (45.5%)	14 (42.4%)	
Severe (30–49)	5 (15.2%)	1 (3.0%)	

SD—Standard Deviation; FEV—Forced Expiratory Volume.

**Table 3:** Bronchoalveolar lavage fluid analysis.

Variables	Malignant (*n* = 33)	Benign (*n* = 33)	*p*-Value
**Multiplex PCR assay**			
Commensal flora	27 (81.8%)	30 (90.9%)	0.281
*Pseudomonas aeruginosa*	3 (9.1%)	0 (0.0%)	0.076
*Streptococcus* spp.	3 (9.1%)	4 (12.1%)	0.689
*Acinetobacter* spp.	5 (15.2%)	0 (0.0%)	0.002
*Klebsiella* spp.	6 (18.2%)	5 (15.2%)	0.741
*Staphylococcus aureus*	3 (9.1%)	0 (0.0%)	0.076
*Escherichia coli*	8 (24.2%)	6 (18.2%)	0.547
*Candida* spp.	2 (6.1%)	8 (24.2%)	0.039
*Serratia* spp.	0 (0.0%)	2 (6.1%)	0.150
Parainfluenza virus	4 (12.1%)	0 (0.0%)	0.039
Other viruses	0 (41.8%)	0 (41.4%)	-
**Culture media**			
Commensal flora	25 (75.8%)	26 (90.9%)	0.768
*Pseudomonas aeruginosa*	2 (6.1%)	0 (0.0%)	0.150
*Streptococcus* spp.	4 (12.1%)	1 (3.0%)	0.162
*Acinetobacter* spp.	5 (15.2%)	0 (0.0%)	0.200
*Klebsiella pneumoniae*	5 (15.2%)	5 (15.2%)	1
*Staphylococcus aureus*	2 (6.1%)	0 (0.0%)	0.150
*Escherichia coli*	5 (15.2%)	6 (18.2%)	0.741
*Candida* spp.	3 (9.1%)	7 (21.2%)	0.169
**Cytology**			
Atypical cells (↑, %)	13 (39.4%)	4 (12.1%)	0.011
Tumoral cells (↑, %)	10 (30.3%)	0 (0.0%)	<0.001
Lymphocytes (↑, %)	8 (24.2%)	23 (69.7%)	<0.001
Neutrophils (↑, %)	9 (27.3%)	5 (12.1%)	0.121
Eosinophils (↑, %)	0 (36.4%)	6 (51.7%)	0.010

IFN—Interferon; IL—Interleukin; TNF—Tumor Necrosis Factor; PCR—Polymerase Chain Reaction.

**Table 4:** Scale and survey results.

Variables	Malignant (*n* = 33)	Benign (*n* = 33)	*p*-Value
**At diagnosis**			
ECOG (mean ± SD)	2.34 ± 0.66	1.92 ± 0.24	0.001
Karnofsky (mean ± SD)	71.36 ± 8.68	79.34 ± 6.18	<0.001
GAD-7 (mean ± SD)	7.18 ± 2.41	5.45 ± 3.52	0.023
PHQ-9 (mean ± SD)	4.90 ± 2.29	3.45 ± 2.84	0.026
Follow-up *			
ECOG (mean ± SD)	2.08 ± 0.52	1.71 ± 0.39	0.002
Karnofsky (mean ± SD)	75.12 ± 8.07	82.60 ± 6.49	<0.001
GAD-7 (mean ± SD)	6.61 ± 2.25	5.07 ± 3.14	0.035
PHQ-9 (mean ± SD)	4.27 ± 2.06	3.91 ± 1.95	0.468

*—The follow-up was performed 90 days after the initial presentation; SD—Standard Deviation; ECOG—Eastern Cooperative Oncology Group performance status (higher scores are associated with lower performance status); Karnofsky scale—higher scores represent a better functional status; GAD—General Anxiety Disorder (higher scores indicate more severe anxiety symptoms); PHQ—Patient Health Questionnaire (higher scores indicate more severe depression symptoms).

**Table 5:** HADS questionnaire results.

Variables	Malignant (*n* = 33)	Benign (*n* = 33)	*p*-Value
**At diagnosis**			
Anxiety (mean ± SD)	7.6 ± 4.1	6.7 ± 3.5	0.341
Depression (mean ± SD)	7.2 ± 3.6	5.3 ± 2.3	0.013
Total score (mean ± SD)	12.8 ± 6.3	10.1 ± 4.2	0.044
Follow-up *			
Anxiety (mean ± SD)	6.9 ± 4.7	6.2 ± 4.5	0.538
Depression (mean ± SD)	6.8 ± 3.1	5.1 ± 4.8	0.092
Total score (mean ± SD)	11.4 ± 5.5	9.8 ± 6.1	0.267

*—The follow-up was performed 90 days after the initial presentation; HADS—Hospital Anxiety and Depression Scale (higher scores indicate greater levels of anxiety or depression).

**Table 6:** The association between bronchial microbiota and depression, as measured on the PHQ-9 survey.

Variables	OR	95% CI	*p*-Value
*Pseudomonas aeruginosa*	1.1	0.8–1.4	0.662
*Streptococcus* spp.	0.9	0.7–1.2	0.450
*Acinetobacter* spp.	1.2	0.9–2.0	0.214
*Klebsiella* spp.	1.0	0.7–1.3	0.896
*Staphylococcus aureus*	1.5	0.8–2.2	0.091
*Escherichia coli*	0.9	0.5–1.4	0.270
*Candida* spp.	1.4	0.9–2.7	0.106
*Serratia* spp.	0.6	0.5–1.1	0.742
Parainfluenza virus	1.7	0.7–2.9	0.060

OR—Odds Ratio; CI—Confidence Interval.

## Data Availability

Data available on request.
